# Peptidoglycan architecture dictates protein interactions, tissue tropism, and arthritis in the Lyme disease spirochete *Borrelia burgdorferi*

**DOI:** 10.1371/journal.ppat.1013849

**Published:** 2026-01-20

**Authors:** Saadman S. Ahmad, Osamudiamen Ebohon, Mecaila E. McClune, Rebecca N. Trimble, Casey N. Kellogg, Carmen J. Booth, Wolfram R. Zückert, Brandon L. Jutras

**Affiliations:** 1 Department of Microbiology-Immunology, Feinberg School of Medicine, Northwestern University, Chicago, Illinois, United States of America; 2 Department of Biochemistry, Virginia Tech, Blacksburg, Virginia, United States of America; 3 Center for Emerging, Zoonotic, and Arthropod-borne Pathogens, Virginia Tech, Blacksburg, Virginia, United States of America; 4 Human Center for Immunobiology, Feinberg School of Medicine, Northwestern University, Chicago, Illinois, United States of America; 5 Department of Comparative Medicine, Yale University, School of Medicine, New Haven, Connecticut, United States of America; 6 Department of Microbiology, Molecular Genetics and Immunology, University of Kansas School of Medicine, Kansas City, Kansas, United States of America; Centre National de la Recherche Scientifique, FRANCE

## Abstract

Lyme disease is a vector-borne illness transmitted by infected *Ixodes spp.* ticks. Dissemination of the Lyme spirochete—*Borrelia burgdorferi—*from the tick bite site results in a bi-phasic infection; the latter phase can cause severe musculoskeletal disease including arthritis. Lyme arthritis is an inflammatory disorder and maladaptive immune response to *B. burgdorferi* infection and its cellular products. One such product, which has been implicated as a key mediator of Lyme arthritis, is peptidoglycan. Peptidoglycan (PG) is a near ubiquitous feature of the bacterial cell envelope, but several chemical features make *B. burgdorferi* PG distinct from other members of the kingdom. We hypothesized the overall chemical composition and structural architecture of the *B. burgdorferi* cell wall are essential to Lyme disease pathogenesis. To manipulate the PG peptide chemical composition, as well as the native cross-links, we produced an isogenic deletion of a putative PG carboxypeptidase *dacA* homologue and assessed both the molecular and cellular phenotypes while probing the pathogenicity of our mutant strain. Our combined and comprehensive approach indicates while changes to PG stem peptide and cross-linking have virtually no discernable impact on any *B. burgdorferi* characteristic in vitro, alterations have significant impacts on tissue tropism and result in a near complete attenuation of Lyme arthritis. PG sacculi containing increased amounts of free and cross-linked pentapeptide surprisingly caused the disassociation of p83/100, an abundant periplasmic protein of unknown function previously implicated in joint tropism, likely contributing to a marked decrease in pathogenicity. These studies strengthen our understanding of the *B. burgdorferi* cell envelope, its unusual components, and further define bacterial features that mediate infectious arthritis.

## Author summary

Many of the pathogenic mechanisms by which *Borrelia burgdorferi* causes disease involve cell envelope interactions with host components. To further elucidate the role of peptidoglycan in Lyme disease pathology we assessed a mutant strain with altered proportions of glycopeptides and found a near complete attenuation of arthritis. Cellular and molecular studies indicate this phenotype could be partially explained by disrupting the interactions between peptidoglycan and a previously uncharacterized periplasmic protein known to impact tissue tropism. Our findings suggest that the complex cell wall of *B. burgdorferi,* and its associated factors, are important contributors to Lyme disease pathogenesis.

## Introduction

Lyme disease (or Lyme Borreliosis) is one of the most reported bacterial infections in the United States [1]. Several biotic and abiotic factors have driven the expansive increase in both case numbers and geographical region of the tick-borne illness [2]. While the increased prevalence in recent years has garnered considerable attention from physicians, patients, and advocates alike, genetic evidence from historic remains coupled with phylogenetic reconstructions collectively suggest that humans have dealt with the consequences of Lyme disease for centuries [3,4]. All past, present, and future projections predict Lyme disease will continue to plague human health.

Clinical manifestations of Lyme disease are diverse and multifactorial. The acute stage of disease occurs within days of spirochete transmission from an infected *Ixodes spp.* tick [5]. Apart from skin rash(es) that may occur in some patients, initial symptoms are often vague and mimic many other illnesses. Without proper intervention, a disseminated infection will occur within weeks to months [6]. These later stages of disease can cause heart and neurological complications, but the most common illness in the United States is arthritis [5,7]. Inherent host factors, along with bacteriological products that may influence pathogenicity and/or tissue tropism, are thought to predicate both the disease presentation and severity [8].

Many of the typical virulence factors that pathogens produce to cause disease are missing from the *Borrelia burgdorferi* genome, the primary agent of Lyme disease in North America. For instance, classic effectors, and the machinery necessary to deploy them, are not thought to be produced by *B. burgdorferi* [9]. Both specialized toxins and those considered more broadly conserved in diderms (e.g., lipopolysaccharide/endotoxin) are also conspicuously absent from *Borrelia* species and strains known to cause human Lyme disease [10–12]. Thus, it is likely that basic cellular products associated with the unique bacterium’s tick vector-vertebrate host life cycle also act as virulence factors in susceptible hosts and tip the balance towards a particular clinical presentation.

The peptidoglycan (PG) of *B. burgdorferi* has recently been implicated as one such factor which could potentiate a robust and maladaptive immune response in certain hosts [13–15]. PG is a hallmark of virtually all bacterial cell envelopes where it acts as both a loadbearing structure and osmoprotectant, but it has also been implicated in pathogenesis where it may act as a virulence factor by modulating host responses in several instances [16–18]. Not only is *B. burgdorferi* PG released during growth where it may interact with host immune cells, but its chemical properties are unique [13]. For example, unlike most bacteria, the diamine involved in cross-linking peptides to adjacent glycan strands is L-Ornithine [13,19,20]. Alterations to the aforementioned glycan strands are also apparent since *Borrelia spp.* produce PG with a disaccharide of *N-*acetyl-glucosamine which is thought to contribute to the exceptionally long half-life of *B. burgdorferi* PG in mouse models of Lyme disease infection [14,20]. Lessons from mouse studies may extend to human Lyme arthritis since patients possess detectable levels of the cell wall biopolymer in the synovial fluid of their swollen joints [13,14]. How these peculiarities impact the overall severity and/or outcome of a *B. burgdorferi* infection remains poorly understood.

We postulated that alterations to the overall chemical architecture of *B. burgdorferi* PG may impact the host response and thus pathogenesis. PG is, however, essential and gross perturbations to salient features in the *B. burgdorferi* cell wall are unlikely to be tolerated. Here, we describe and characterize a mutant strain of *B. burgdorferi* with altered cell wall properties where the nascent sacculus possesses exceptionally high levels of non-cross-linked pentapeptide. Despite behaving nearly identical to a parental control strain in vitro, in vivo studies using a mouse model of infection suggest a near complete attenuation of Lyme arthritis. To better understand this response, we analyzed the *B. burgdorferi* cell envelope of our mutant and discovered that p83/100—an abundant periplasmic protein implicated in joint tropism—was significantly diminished in its previously unknown role in PG binding. Our findings provide new insights into *B. burgdorferi* physiology and pathogenesis, including a previously unappreciated relationship between an immunodominant antigen and the Lyme spirochete sacculus.

## Results

### Characterizing the function of BB0605 in *B. burgdorferi* physiology

We sought to understand how the chemical and architectural properties of *B. burgdorferi* PG may impact Lyme disease pathogenesis. Mur enzymes are essential intracellular proteins dedicated to producing the PG precursor(s) necessary for de novo cell wall (or murein) synthesis and as such, cannot be easily inactivated or altered by genetic manipulation. On the other hand, transpeptidase and transglycosylase enzymes involved in incorporating PG sub-units, and in modifying the existing sacculus, are dispensable under certain environmental conditions [21–23]. We reasoned that this may be the case for *B. burgdorferi* as well, so we mined a previously published transposon mutant library for integration events that may disrupt transpeptidase and transglycosylase reading frames [24]. Two such clones had transposon integrations at nucleotide positions 152 and 300, relative to the beginning of the *bb0605* gene*.* BB0605 is predicted to encode D-Ala-D-Ala carboxypeptidase since it carries common features shared by PBP5/DacA family orthologues, including 1) a membrane bound, extended amphipathic helix C- terminus; 2) N-terminus that lacks a helix; and 3) a loop shielding the catalytic Serine residue (S1A–S1D Fig, and [25]).

**Fig 1:**
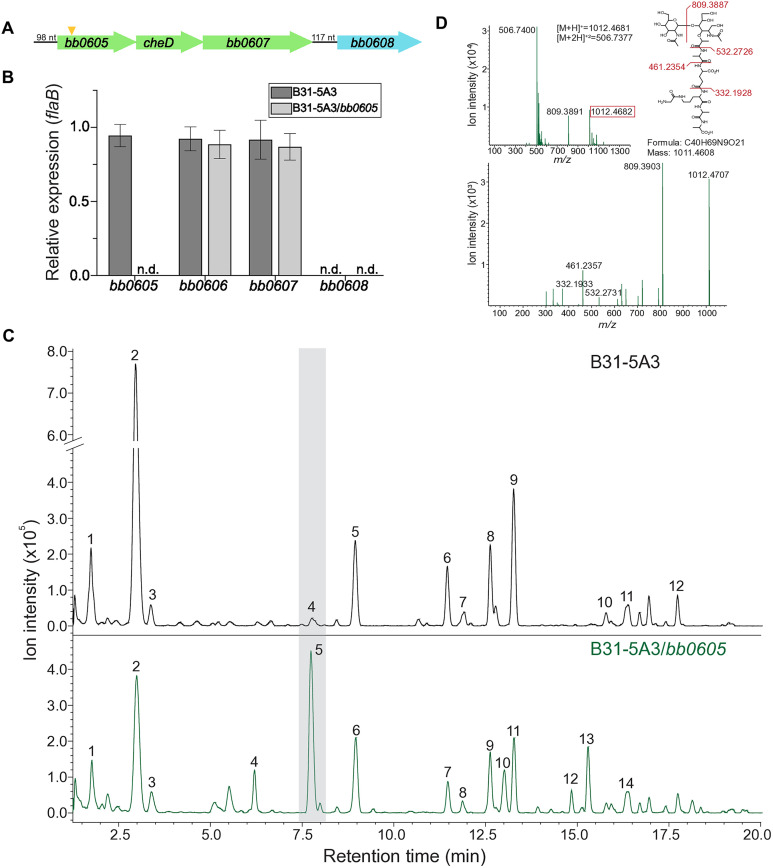
Strain validation and LCMS analysis of PG isolated from B31-5A3/*bb0605.* **(A)** Schematic of the putative *bb0605* operon, as well as the downstream *bb0608* locus, mapped from the *B. burgdorferi* B31 type strain genome. The transposon integration site (+300, 5’ to 3’) in *bb0605* is shown (triangle) as are the length, and location, of predicted non-coding regions. **(B)** Relative mRNA levels of target loci to validate B31-5A3/*bb0605* mutant. RT-PCR was performed on RNA isolated from both parental (B31-5A3, charcoal) and mutant (B31-5A3/*bb0605,* light gray) strains using locus-specific primers and normalized relative to constitutively expressed *flaB.* Values are the mean + /- SD. n.d., not detected after 45 cycles. **(C)** Identifying changes in the peptidoglycan composition of B31-5A3/*bb0605*. Liquid chromatogram of mutanolysin treated PG isolated from B31-5A3 (top, in black) and B31-5A3/*bb0605* (bottom, in green). Each numbered peak corresponds to a muropeptide found in Tables 1 and 2, respectively. Peak 5 (shaded gray) corresponds to the muropeptide Glc*N*Ac-Mur*N*Ac-Ala-Glu-Orn-[Gly]-Ala-Ala). **(D)** MS (top) and MS/MS (bottom) of the Glc*N*Ac-Mur*N*Ac-Ala-Glu-Orn-[Gly]-Ala-Ala muropeptide. MS/MS fragmentation (inset) data confirms the pentapeptide(-Gly) structure.

To understand the potential role for *bb0605* in *B. burgdorferi* PG biosynthesis we sought to create an isogenic mutant through typical gene disruption methods [26], but all attempts failed for unknown reasons. Instead, we took an alternative approach whereby the *B. burgdorferi* 5A18NP1 *bb0605*::Himar*-1* transposon mutant genomic DNA was used as a template for PCR using primers which flanked the target locus by ~2kb. This amplicon was sub-cloned, the resulting plasmid was linearized and then used to displace the *bb0605* locus in the *B. burgdorferi* B31-5A3 genetic background by allelic exchange. The *bb0605* locus lies in a putative operon with genes encoding for CheD and a predicted DNA helicase (BB0607, Fig 1A). To assess our new strain, and understand any potential polar effects, we isolated RNA and compared mRNA levels to a parental, *B. burgdorferi* B31-5A3 control strain [27]. Replacement of the targeted locus with the *Himar-1* transposase knockout construct resulted in undetectable amounts of *bb0605*, while levels in the wild-type strain were comparable to the highly expressed *flaB* (Fig 1B)*.* Importantly, mRNA levels of the adjacent loci (i.e., *cheD* and *bb0607*) were unaffected by our genetic manipulation (Fig 1A and 1B). Transcriptomic analysis of the operon suggested complete disruption of *bb0605* expression, without impacting other loci, but we confirmed both the integration site (after nucleotide 299 of the gene) and the absence of distal polar mutations in coding regions of our mutant strain using whole genomic sequencing (WGS) (S1 Table). Together, we contend our new strain (*B. burgdorferi* B31-5A3/*bb0605*) is incapable of synthesizing *bb0605* and WGS suggest the mutant is free of any unintended mutations.

**Table 1:** Muropeptide analysis of peptidoglycan purified from *B. burgdorferi* B31-5A3.

Peak #	Muropeptide ID	[M+H]^+^	Structure	RT (min)
1	1	667.3148	Mur*N*Ac-Ala-Glu-Orn-Gly	1.753
2	2a	870.3942	Glc*N*Ac-Mur*N*Ac-Ala-Glu-Orn-Gly	2.928
3	2b	870.3942	Glc*N*Ac-Mur*N*Ac-Ala-Glu-Orn-Gly	3.385
4	3	1012.4669	Glc*N*Ac-Mur*N*Ac-Ala-Glu-Orn-[Gly]-Ala-Ala	7.757
5	4a	850.3670	Glc*N*Ac-Mur*N*AcAnh-Ala-Glu-Orn-Gly	8.945
	5	1053.4462	Glc*N*Ac-Glc*N*Ac-Mur*N*AcAnh-Ala-Glu-Orn-Gly	
6	4b	850.3668	Glc*N*Ac-Mur*N*AcAnh-Ala-Glu-Orn-Gly	11.455
7	7	1386.6478	Mur*N*Ac-Ala-Glu-Orn-[Gly]-Ala-Gly-Orn-Glu-Ala-Mur*N*Ac	11.883
8	8	1589.7249	Glc*N*Ac-Mur*N*Ac-Ala-Glu-Orn-[Gly]-Ala-Gly-Orn-Glu-Ala-Mur*N*Ac	12.623
9	9	1792.803	Glc*N*Ac-Mur*N*Ac-Ala-Glu-Orn-[Gly]-Ala-Gly-Orn-Glu-Ala-Mur*N*Ac-Glc*N*Ac	13.250
10	10	1975.8633	Glc*N*Ac-Glc*N*Ac-Mur*N*AcAnh-Ala-Glu-Orn-[Gly]-Ala-Gly-Orn-Glu-Ala-Mur*N*Ac-Glc*N*Ac	15.780
11	11a	1772.7758	Glc*N*Ac-Mur*N*Ac-Ala-Glu-Orn-[Gly]-Ala-Gly-Orn-Glu-Ala-Mur*N*AcAnh-Glc*N*Ac	16.377
12	11b	1772.7766	Glc*N*Ac-Mur*N*Ac-Ala-Glu-Orn-[Gly]-Ala-Gly-Orn-Glu-Ala-Mur*N*AcAnh-Glc*N*Ac	17.728

**Table 2:** Muropeptide analysis of peptidoglycan purified from *B. burgdorferi* B31-5A3/*bb0605.*

Peak #	Muropeptide ID	[M+H]^+^	Structure	RT (min)
1	1	667.3148	Mur*N*Ac-Ala-Glu-Orn-Gly	1.765
2	2a	870.3942	Glc*N*Ac-Mur*N*Ac-Ala-Glu-Orn-Gly	2.990
3	2b	870.3942	Glc*N*Ac-Mur*N*Ac-Ala-Glu-Orn-Gly	3.392
4	3	809.3890	Mur*N*Ac-Ala-Glu-Orn-[Gly]-Ala-Ala *	6.202
5	4	1012.4681	Glc*N*Ac-Mur*N*Ac-Ala-Glu-Orn-[Gly]-Ala-Ala *	7.740
6	5a	850.3678	Glc*N*Ac-Mur*N*AcAnh-Ala-Glu-Orn-Gly	8.963
	6	1053.4473	Glc*N*Ac-Glc*N*Ac-Mur*N*AcAnh-Ala-Glu-Orn-Gly	
7	5b	850.3678	Glc*N*Ac-Mur*N*AcAnh-Ala-Glu-Orn-Gly	11.475
8	7	1386.6484	Mur*N*Ac-Ala-Glu-Orn-[Gly]-Ala-Gly-Orn-Glu-Ala-Mur*N*Ac	11.872
9	8	1589.7268	Glc*N*Ac-Mur*N*Ac-Ala-Glu-Orn-[Gly]-Ala-Gly-Orn-Glu-Ala-Mur*N*Ac	12.628
	9	1195.5211	Glc*N*Ac-Glc*N*Ac-Mur*N*AcAnh-Ala-Glu-Orn-[Gly]-Ala-Ala *	
10	10	992.4420	Glc*N*Ac-Mur*N*AcAnh-Ala-Glu-Orn-[Gly]-Ala-Ala *	13.008
11	11	1792.8059	Glc*N*Ac-Mur*N*Ac-Ala-Glu-Orn-[Gly]-Ala-Gly-Orn-Glu-Ala-Mur*N*Ac-Glc*N*Ac	13.277
12	12	1731.7998	Glc*N*Ac-Mur*N*Ac-Ala-Glu-Orn-[Gly]-Ala-Gly-[Ala-Ala]-Orn-Glu-Ala-Mur*N*Ac *	14.848
13	13	1934.8796	Glc*N*Ac-Mur*N*Ac-Ala-Glu-Orn-[Gly]-Ala-Gly-[Ala-Ala]-Orn-Glu-Ala-Mur*N*Ac-Glc*N*Ac *	15.292
14	14	1772.7772	Glc*N*Ac-Mur*N*Ac-Ala-Glu-Orn-[Gly]-Ala-Gly-Orn-Glu-Ala-Mur*N*AcAnh-Glc*N*Ac	16.373

Note: * indicates a muropeptide that is unique to the *bb0605* mutant strain.

The *B. burgdorferi* genome is riddled with anomalies and functional inferences made purely on silico homology have been misleading in the past [28–30]. As such, we assessed the PG composition of the *bb0605* mutant using our recently described high-resolution LCMS method [20]. Our analysis revealed disaccharide-pentapeptide(-Gly)—a structure nearly undetectable in wild-type bacteria—became one of the most predominant muropeptide species in BB0605-deficient bacteria, which we confirmed by reconstructing the fragmentation pattern in MS/MS spectra (Fig 1C and 1D). Additionally, high-molecular weight PG peaks corresponding to cross-linked species were altered in terms of their retention time and abundance (Fig 1C). The latter suggested the formation of new cross-linked species. Further mining of MS/MS data identified many instances in which the new D-Ala-D-Ala species were now present in cross-linked muropeptides, while the same structures did not exist in wild-type PG (Tables 1 and 2). Surprisingly, the degree to which muropeptides were cross-linked in each strain did not change (~33%), but di-D-Ala-containing muropeptides were now present in ~55% of the total amount of peptide bridges (S2 Table). These findings strongly suggest that BB0605 is a functional D-Ala-D-Ala carboxypeptidase. Most importantly, our mutant strain was altered in both PG peptide composition and the type of cross-linked species present in the nascent *B. burgdorferi* sacculus. Given the homology (S1B–S1D Fig), and the clear biochemical properties of the native enzyme in the *B. burgdorferi* cell, we will refer to BB0605 as DacA from here forward.

**Fig 2:**
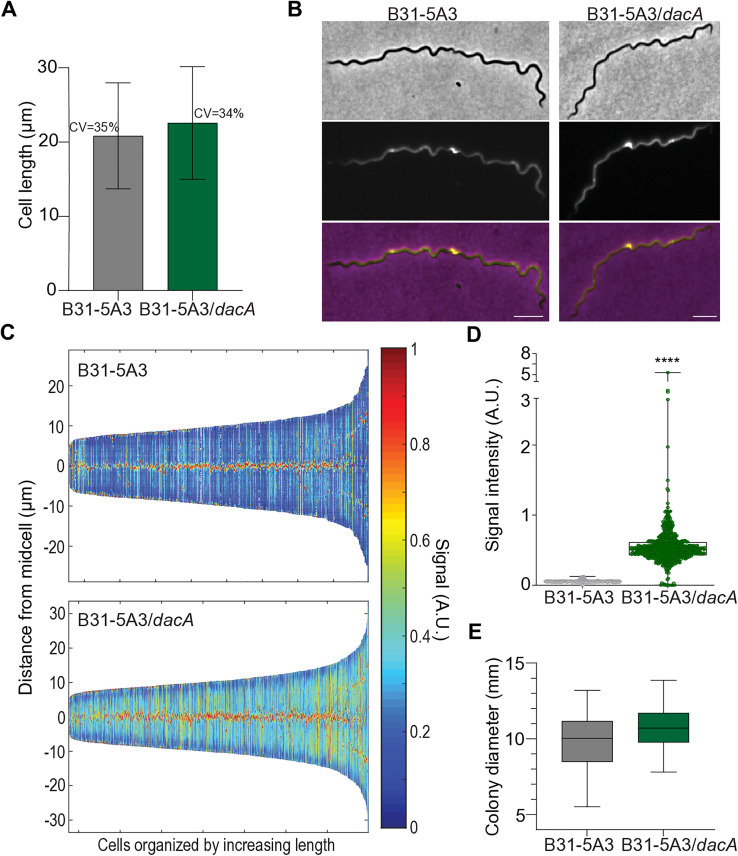
Cell cycle and PG synthesis analysis of the carboxypeptidase mutant *dacA.* **(A)** Cell length analysis as a proxy for defects in cell cycle progression. *B. burgdorferi* B31-5A3 (gray, *n *= 410) and B31-5A3/*dacA* (green, *n *= 724) strains were cultured to mid-log exponential growth, imaged by phase-contrast microscopy, and micrograph data collected was analyzed with Oufti to determine the length of each cell in the population with sub-pixel resolution [88]. Shown are the mean + /- SD, as well as the coefficient of variation (CV). **(B)** Analysis of zonal PG synthesis. Phase-contrast (upper), Epifluorescence (middle), and merge (lower) images are shown for both strains incubated with 0.25 mM HADA for 4 hrs. Scale bar = 5 µm. **(C)** Demograph of HADA signal intensity derived from *B. burgdorferi* B31-5A3 parental (top, *n *= 410) and B31-5A3/*dacA* mutant (bottom, *n *= 724) strains, attained from individual cells, organized by length. Signal intensity is shown in arbitrary units (A.U) as a heatmap. **(D)** Population-level HADA fluorescent signal intensity analysis of data collected in C, *B. burgdorferi* B31-5A3 (gray) and B31-5A3/*dacA* (green). **** *p *< 0.0001, unpaired Student’s t-test. **(E)** Swarm assay to assess motility. A single soft agar plate was inoculated with 7.5 µL of 10^9^ cells/mL suspensions of both strains *B. burgdorferi* B31-5A3 (gray) and B31-5A3/*dacA* (green), on opposite sides, and colony diameters were measured after 5 days. Data shown are the mean of 3 replica plates, + /- SD. Statistical significance was assessed using an unpaired Student’s t-test.

PG synthesis is intimately linked to all aspects of the bacterial cell cycle in most bacteria [31–34]. Dysregulation of any major cell cycle event usually results in gross morphological and/or physiological phenotype(s) [22,35–37]. To assess the overall physiological state of our mutant, we used proxies for cell cycle and cell envelope homeostasis and compared our results to the parental strain. We first assessed the growth by back-diluting cultures and manually enumerated cells daily. Despite exhibiting a longer lag phase, the exponential growth rate of the *dacA* mutant was slightly faster than the parental control (10.8 vs. 13.6 hours) and both reached a similar final density (S1E Fig). We also assessed both the cell length and the coefficient of variation (CV), which were almost identical: parental CV 35%; mutant CV 34% (Fig 2A). CV of cell length is a proxy for cell cycle homeostasis and can be affected by PG alterations [32,35,38–41]. We further assessed the possible impact of the carboxypeptidase mutant on the *B. burgdorferi* cell cycle by quantitative analysis of PG synthesis. Analysis of cells pulse-labeled with fluorescent D-Ala, in conjunction with real-time localization studies of known divisome proteins indicate *B. burgdorferi* elongates by synthesizing discrete zones of PG that are equally spaced and become future sites of cell division [42,43]. We repeated the former and assessed the relative sub-cellular locations of PG synthesis in both strains throughout the cell cycle (i.e., from cell birth to division) and found that the absence of DacA did not affect the position of zonal growth sites (Fig 2B and 2C). During these studies we noticed the fluorescent signal intensity appeared to be higher in the mutant (Fig 2C). We quantified the fluorescent signal from each population by determining the intensity of each pixel in each cell, calculated the average, and normalized by area for hundreds of cells and found the signal in the *dacA* mutant was ~ 5-fold higher (Fig 2D). Our interpretation of these collective results is that the *B. burgdorferi* cell cycle is not altered in the absence of DacA and that fluorescent signal intensity differences are likely due to the terminal D-Ala (i.e., where HADA is incorporated) not being processed by the D-Ala-D-Ala carboxypeptidase.

Motility is essential to *B. burgdorferi* infectivity and pathogenesis [44]. Given the conceptual and biophysical relationship between the periplasmic flagella and *B. burgdorferi* PG [20,45,46], we considered the possibility that our mutant strain with altered PG organization may have motility defects. We did not detect any significant difference between strains using swarm plate assays, suggesting the PG peptide composition and linkage status did not alter the intimate relationship between the two distinct but related *B. burgdorferi* ultra-structures (Fig 2E). Together, our mutant strain characterization indicates that the *B. burgdorferi* DacA homologue is a bona fide terminal PG peptide carboxypeptidase, and that alterations created as a result of its inactivation do not produce gross cellular or physiological defects (Figs 1, 2, and S1E).

### Altering PG peptides attenuates Lyme arthritis

**Fig 3:**
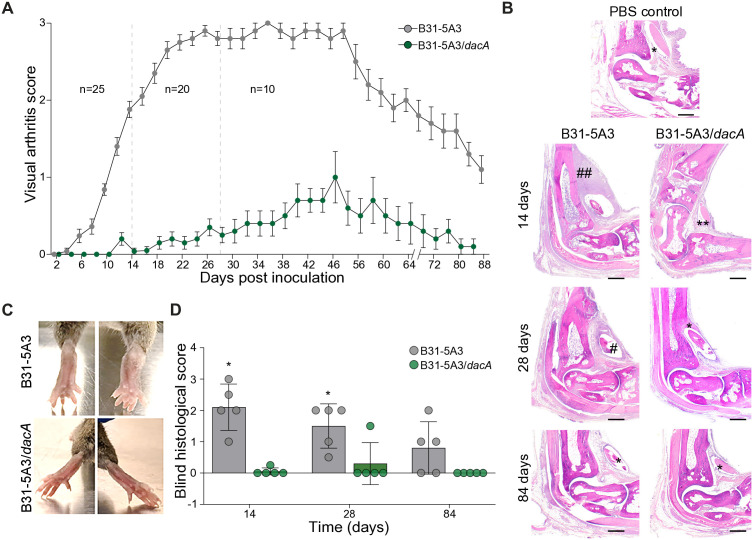
*dacA* mutant bacteria do not cause arthritis in a murine model of Lyme disease. Mice injected with B31-5A3/*dacA* exhibit severely attenuated Lyme arthritis. (**A**) Both wild-type B31-5A3 and B31-5A3/*dacA* bacteria were needle inoculated (10^5^ cells) into C3H/HeJ mice (*n *= 25 per group), and arthritis severity was assessed by visual arthritis scoring every two days. Two and four-weeks post injection, five and ten animals, respectively, were sacrificed (dotted lines) for histopathological analysis and qPCR (see Figs 4 and S4). Mean + /- SEM are shown. (**B**) Representative histopathology of HE-stained sections of tarsal joints from mice infected with *B. burgdorferi* B31-5A3 (left) and B31-5A3/*dacA* (right) 14-, 28-, and 84-days post injection. A single PBS control tissue is also shown. Arthritis scores for tissues shown in this panel were as follows: * = 0.0, ** = 0.25, # = 1.0, ## = 2 – 3. (**C**) Representative ankles from C3H/HeJ mice infected with *B. burgdorferi* B31-5A3 (upper) or B31-5A3/*dacA* (lower), 4-weeks after infection. (**D**) Blind histopathological scores for individual mice in each group at 14-, 28-, and 84--days post PG administration. Statistical significance was determined using Welch’s t-test, * *p *≤ 0.05 (*).

*B. burgdorferi* PG has been implicated in the development of Lyme arthritis in both mice and humans [13,14]. To assess how changes to PG structure may impact the former, we needle inoculated a cohort of C3H mice with either the *dacA* mutant or parental control strain (10^5^ cells) and tracked arthritis severity for > 12 weeks. C3H were selected because of their inherent capacity to develop severe murine Lyme arthritis [47–49]. Wild-type *B. burgdorferi* B31-5A3 produced visible arthritis in C3H mice within 2 weeks post infection, while the *dacA* mutant appeared to lag (Fig 3A and 3B). Five mice were sacrificed at this time point and while subtle differences were apparent in bacterial load, as assessed by qPCR, none were statistically significant (S2 Fig). Wild-type infected mice continued to experience marked arthritis, which peaked 28 days after inoculation and remained apparent for at least 60 days post-infection by visual metrics (Fig 3A–3C). Blind histopathological analysis—a more quantitative assessment of arthritis—was consistent with gross visual arthritis scoring throughout wild-type infection (Fig 3B and 3D). Infection with mutant bacteria unable to produce DacA, on the other hand, produced undetectable levels of murine Lyme arthritis in virtually all mice (Fig 3). A single animal (4%) appeared to have histological evidence of arthritis, but severity did not increase in the population of mice infected with *B. burgdorferi* B31-5A3/*dacA* and remained near basal levels even after 12 weeks (Figs 3A, 3B, 3D, and S3).

**Fig 4:**
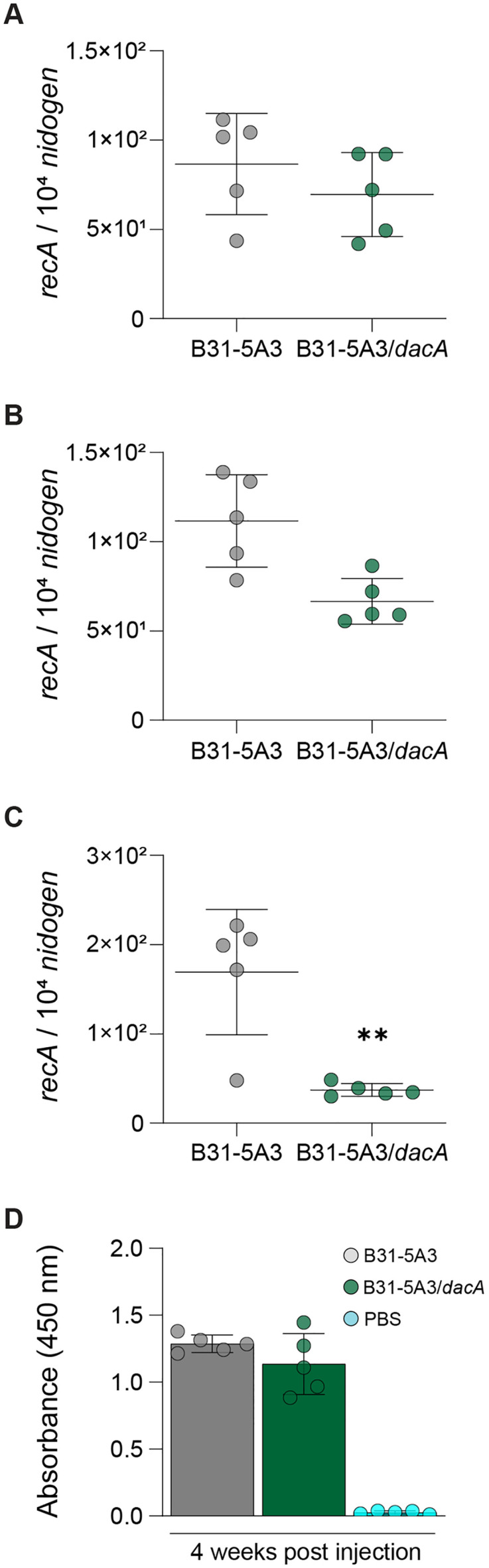
Alterations to PG peptides and cross-links affect tissue tropism. **(A)** qPCR analysis of heart **(A),** ear **(B)**, and ankle **(C)** tissue 4-weeks post injection. The number of copies of *recA* were quantified using a standard curve and then normalized to murine *nidogen*. The black bars indicate the average normalized bacterial load for each group of tissues, + /- SD. Statistical significance was calculated using an unpaired Student’s t-test *p *≤ 0.01 (**). **(D)** IgG response to infection. Total, *B. burgdorferi-*specific IgG levels were quantified by incubating plasma samples from mice injected with B31-5A3, B31-5A3/*dacA* or PBS in 96-well microplates coated with *B. burgdorferi* lysate and determining absorbance at 450 nm.

Disseminated stages of Lyme disease in mice produces a multi-system infection whereby bacteria can be cultured from, and their products detected in, various tissues [50,51]. We first determined that the significant attenuation of murine Lyme arthritis was not simply due to the *dacA* mutant strain being non-infectious, which was confirmed by culturing live bacteria from the bladders of all mice, infected with both strains (Table 3). We returned to qPCR of select tissues to further understand the arthritis phenotype and determine if bacterial load was playing a role. We focused on three tissues that frequently harbor spirochetes in murine Lyme disease—heart, ankle, and skin. The latter was assessed in ear tissue, which is distal relative to the sub-cutaneous site of mouse inoculation (rear flank) and thus an excellent measure of bacterial dissemination. Four-weeks post infection, both skin and heart tissues from mice infected with both strains contained comparable levels of *B. burgdorferi* DNA (Fig 4A and 4B). Quantitative assessment of ankle tissues, however, resulted in a modest, but significantly lower bacterial burden in animals infected with the *dacA* mutant (Fig 4C). Total IgG levels at this stage of infection were similar (Fig 4D). We also assessed ankle tissues 12 weeks post infection, a point at which arthritis was still visible in wild-type infected animals and found no difference between strains (S4 Fig). These data suggest PG composition and overall architecture are important determinants of Lyme arthritis, findings that may be partially explained by playing direct, or indirect, role(s) in tissue tropism.

**Table 3:** Organ outgrowth from infected mice sacrificed 4 weeks post-infection.

Strain	Organ	Weeks post inoculation	Outgrowth outcome
B31-5A3	Bladder	4	5/5
B31-5A3/*dacA*	Bladder	4	5/5

### p83/100 antigen is a peptidoglycan-associated protein

**Fig 5:**
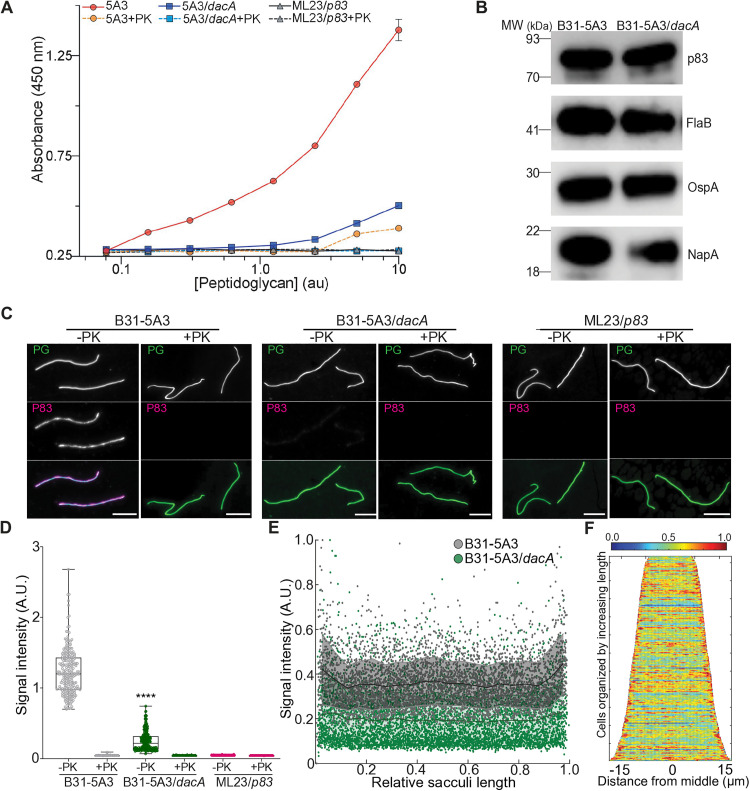
Immunodominant antigen p83/100 is a PG-associated protein (PAP) whose interactions are impacted by alterations in cell wall chemistry. **(A)** ELISA-based approach to assess PG-p83/100 interaction(s). Sacculi from *B. burgdorferi* B31-5A3, B31-5A3/*dacA*, B31-ML23/*p83*/100 strains were purified and one-half was treated with proteinase K (+PK) while the other was incubated with buffer (-PK). A serial titration of each purified PG preparation was immobilized on microtiter plates using r-mAb2G10, and the amount of PG and p83/100 was quantified using biotin-r-mAb2G10/streptavidin:HRP, anti-p83/100 coupled with anti-rat:IgG:HRP, respectively. The PG value attained for each strain and preparation was used to normalize each p83/100 value, which were plotted as a function of absorbance (450 nm). **(B)** Lysates from each strain were prepared and total protein loading was optimized using immunoblots with anti-FlaB. After optimization, each immunoblot was performed with the exact same amount of lysate to detect putative PAP (anti-NapA, anti-p83/100), as well as Outer surface protein A (anti-OspA), and the FlaB loading control was repeated (anti-FlaB). **(C)** The same samples described in ‘A’ were used to assess the amount and localization of p83/100 in purified PG. Each PG preparation and treatment were immobilized on optical glass using poly-Lys and probed using PG-binding lectin Wheat Germ Agglutinin conjugated to Oregon Green 488 (PG, green, upper panel) and anti-p83/100 conjugated to anti-rat IgG:Alexa647 (p83/100, magenta, middle panel). A merge of both micrographs described above is also shown using the color scheme described above (lower panel). Scale bar 5 µm. **(D)** Total integrated p83/100 signal intensity for each sample and preparation described in ‘A’ and ‘C’. The signal intensity of each pixel, inside each sacculus was measured, and the average was calculated for each, then normalized by area (one dot). This was repeated for > 100 sacculi per sample and the median of each is shown (black line). One-way analysis of variance was used to determine significance between P83-derived signal present in B31-5A3 wild-type sacculi (-PK), relative to B31-5A3/*dacA* sacculi (-PK), **** *p *< 0.0001, ANOVA. Note that all other comparisons to wild-type (-PK) sacculi were also statistically lower (****) but not shown for simplicity. *n* values for each were as follows: B31-5A3 (-PK), 227; B31-5A3 (+PK), 132; B31-5A3/*dacA* (-PK), 255; B31-5A3/*dacA* (+PK), 133; B31-ML23/*p83*/*100* (-PK), 178; B31-ML23/*p83*/*100* (+PK), 101. **(E)** Population-level line scans of values attained in studies described in ‘C’. P83-derived signal intensity present in purified sacculi from B31-5A3 (-PK, gray) and B31-5A3/*dacA* (-PK, green) was determined by measuring the signal intensity of each pixel along the midline of the cell wall, for each sacculus, and plotted as a function of normalized length (0 – 1). Black lines show the mean of each, SD are shaded. **(F)** Demograph analysis of p83/100 signal intensity in sacculi purified from B31-5A3 (-PK).

PG is often directly or indirectly associated with proteins which 1) provide cell wall stability; 2) secure the position of the sacculus relative to other cell envelope components; 3) ensure continuity between envelope layers; and 4) are occasionally involved in bacterial pathogenesis [52–54]. We previously discovered that Neutrophil Attracting Protein A (NapA) was directly associated with the *B. burgdorferi* PG sacculus and seemingly served many of the functions described above. During our assessment of PG-associated proteins (PAPs) in the Lyme spirochete, apart from NapA, the next most likely PAP candidate was the immunodominant protein p83/100 (BB0744) [15,55–58]. Despite being highly expressed and a serological indicator of infection, little is known about the biological function of p83/100. Intriguingly, mutant bacteria unable to produce p83/100 exhibited defects in tissue tropism and tracking studies in live animals found a paucity of bacteria in infected animal joints [59]. The same study also found that p83/100 localized to the periplasm of *B. burgdorferi*. In the context of the current findings, these data suggested that p83/100 might be a PAP, and that altered PG peptides (e.g., those present in the *dacA* mutant), may impact this association. We therefore assessed this possibility by purifying PG from both wild-type and *dacA* mutant bacteria by boiling each in 5% SDS. After removing SDS we treated half of the PG sacculi preparation with proteinase K (PK) and left the remainder untreated. We also performed the same procedure with a *p83*/*100* mutant strain, as an additional control, and developed an ELISA approach to detect p83/100. A titration of each purified PG preparation was immobilized on microtiter plates using an anti-PG antibody (r-mAb-2G10, [14]), and probed using anti-p83/100 or biotin conjugated r-mAb-2G10. The latter was used to normalize for the total amount of sacculi present in each preparation since slight variations are common due to the lengthy purification process. Purified sacculi isolated from wild-type bacteria contained p83/100-derived fluorescent signal, which increased as the amount of untreated (i.e., PK-) cell wall-containing material increased (Fig 5A). Following protease treatment (PK+), this same sample contained near undetectable levels of p83/100, regardless of the titration (Fig 5A). Purified PG from *dacA* mutant bacteria contained significantly less p83/100 and the p83/100-derived signal returned to basal (i.e., *p83*/*100* mutant) levels after degradation by PK (Fig 5A). Immunoblots with lysates from each strain suggested that cellular levels of p83/100 were comparable and similar to the highly expressed outer surface protein OspA and the FlaB loading control, regardless of DacA production (Fig 5B), indicating that the lower levels of PG-bound p83/100 in the *dacA* mutant is not due to overall expression.

Our initial molecular assessment provided clear evidence that p83/100 is a PAP and alterations to the chemical properties of PG significantly diminish putative cell wall-protein interaction(s) (Fig 5A). We next assessed the same PG samples described above using immunofluorescence to provide additional cellular and spatial context. Sacculi were immobilized on optical glass and probed using anti-p83/100:anti-rat-Alexa647. To prevent any ambiguity in our initial results, we visualized sacculi using fluorescently conjugated Wheat Germ Agglutinin rather than r-mAb2G10. Prior to PK treatment, sacculi isolated from wild-type cells show a clear p83/100 signal, which was barely visible in the *dacA* mutant PG (Fig 5C). To further assess the reduction in signal we measured the intensity of each pixel, inside each sacculus, calculated an average, and normalized the values by total area of the object. We then repeated this procedure, in an automated fashion, for more than 100 sacculi per sample and treatment. Our population-level results suggest that, on average, *dac*A mutant sacculi contain six times less p83/100 (Fig 5D), confirming our initial ELISA results. p83/100 signal distribution was assessed at the population level using quantitative line scans and demographs; both yielded similar results and suggested some periodicity throughout the long axis of the sacculus as well as enrichment at the PG poles (Fig 5E and 5F). In summary, our data indicate that p83/100 is a PAP whose interactions are impacted by PG-peptide composition and both p83/100 and PG together affect bacterial tissue tropism and the development of Lyme arthritis.

## Discussion

Lyme arthritis is an immune-mediated, pathophysiological consequence of *B. burgdorferi* infection. *B. burgdorferi* PG has been implicated as a mediator of the maladaptive response by potentiating sustained inflammation [8,13,14]. One of the many features that differentiate the Lyme disease spirochete cell wall from others are its unusual components, which are likely drivers in arthritis [14]. The initial impetus of this work was to manipulate the overall PG architecture to directly address this scenario in the context of a natural infection, which led to studies on a *dacA* mutant strain. While we were completing this study, McCausland et al. published their findings using the original transposon mutant of *dacA.* Despite differences in the motivation to assess the mutant, as well as the genetic background and the approaches used, they came to the same conclusions in terms of the putative function of BB0605 as a structural homologue of DacA [60]. The same study found *dacA* mutant bacteria have marked increase in penta-peptide-(Gly), produce a central zone of PG synthesis similar to wild-type cells, and exhibit higher signal intensity when incubated with a fluorescent analogue of D-Ala [60]. Robust agreement in the biochemical and cellular data between groups further supports the overall function of BB0605 as a carboxypeptidase and bolsters our additional findings, which we discuss in the context of *B. burgdorferi* physiology and pathogenicity below.

The bacterial kingdom is full of genotypic and phenotypic diversity; production and activity of PBPs contribute to both. Most bacteria produce 10–16 discrete PBPs. In silico and activity-based profiling studies suggest *B. burgdorferi* encodes 4–5 PBPs and DacA is the only predicted type-5 PBP [10,42,43]. PBP5 homologues are known as low molecular weight (LMW) enzymes that typically lack transpeptidase activity, but are often important in controlling cell shape, as well as in ensuring faithful completion of the bacterial cell cycle [25]. For instance, both morphological and cell envelope abnormalities are common in *E. coli* mutants unable to produce DacA [61–64]. Like *B. burgdorferi,* many gram-positive bacteria produce a single PBP5 orthologue, and they too are directly linked to both cell shape and division [65,66]. Morphological variability is attributed to uncoupling PG precursor processing from nascent synthesis leading to osmotic vulnerabilities. Aberrant cell cycle events in carboxypeptidase mutants are thought to be the result of the divisome machinery acting on a surplus of di-D-Alanine-containing PG peptides at the septum [25]. As such, it was surprising to us that *dacA* deficient *B. burgdorferi* 1) did not exhibit a growth defect; 2) appeared morphologically identical to wild-type bacteria; 3) produced properly placed zones of PG synthesis; and 4) formed a viable septum at mid-cell (S1 and S2 Figs). It is important to note that many of the phenotypes associated with PBP5 mutants are often context-dependent and require additional mutations in other PBPs and/or exogenous stress; conditions that may be more reflective of in vivo environments [67].

PG does not appear to play an active role in *B. burgdorferi* shape, but it does counteract deforming torsional stress imparted by endo-flagella wrapping around the cell cylinder [44,68]. Thus, it is thought that the passive function of PG in motility is to provide reciprocal elastic forces that are fine-tuned to create the least amount of drag and allow the flagella to produce the greatest amount of torque [20,46]. Unlike other helical bacteria, whose shape and motility are impacted by carboxypeptidases and cross-linking [69–71], *B. burgdorferi* motility in vitro and dissemination in vivo are unaffected in a *dacA* mutant (Figs 2E, 4A, and 4B). We postulate *B. burgdorferi* has evolved alternative strategies to maintain PG flexibility and continuity that go beyond cross-linking, likely through glycan modifications [20]. It is also possible that maintaining the terminal D-Alanine in cross-bridges do not confer a substantiative biophysical effect but may alter the cell envelope in other ways.

There are numerous examples by which carboxypeptidases are directly responsible for beta-lactam resistance; often through allelic variation and altered cellular concentrations of PBP5 homologues [72–75]. Assessing the true pathogenicity of unprocessed PG stem peptide—resulting in increased pentapeptide and altered cross-links—is challenging because it is difficult to uncouple the fitness cost(s) from the alteration. Yet, some instances are worth noting. Carboxypeptidase Pgp2 is necessary to establish *Campylobacter jejuni* infection in a chick colonization model [71]. DacA-deficient *Streptococcus pneumoniae* remains infectious, but target cell adherence is impaired, and mutant bacteria are more likely to die following phagocytosis [76]. The DacA orthologue in *Staphylococcus aureus*, LMW PBP4, is essential for invasion and colonization of osteocytes, a pre-requisite to causing osteomyelitis, likely by altering the proinflammatory capacity of *S. aureus* PG [77]. It appears *B. burgdorferi* DacA may be functionally similar to *S. aureus* PBP4 in the context of pathogenesis, since similar sacculi changes result in the near complete attenuation of Lyme arthritis, a pathological manifestation of Lyme disease to which PG has been directly linked (Figs 1 and 3, and [13,14]).

We found p83/100—a periplasmic protein of unknown function, also known as BB0744—is a PAP and the carboxypeptidase activity of *B. burgdorferi* DacA directly or indirectly impacted the PG-protein interaction. Evidence for p83/100 interactions with *B. burgdorferi* PG were initially provided in an unbiased proteomics approach [15]. We serendipitously returned to these published data when we observed apparent tissue tropism in the *dacA* mutant strain (Fig 4 and see below) and critically assessed p83/100-PG interactions through molecular and cellular approaches (Fig 5). Further support for this association is provided by earlier studies in which p83/100, like PG and PAP NapA, is also present in *B. burgdorferi* outer-membrane vesicles [15,78]. PG-p83/100 complexes are near ubiquitous throughout the sacculi, with some enrichment at the PG poles, while p83/100-derived signal in intact cells appears uniform (Fig 5E and 5F, and [59]). It is possible that the increased polar signal in purified sacculi reflects the static, PG-linked population of p83/100 in the cell, which is not captured when visualizing the entire periplasmic space. It remains to be determined how p83/100 interacts with the *B. burgdorferi* cell wall, but resistance to boiling SDS treatment suggests a covalent linkage. It is possible that the interaction(s) are non-covalent and could be released with longer SDS treatment, as was the case for LipL21 in *Leptospira interrogans* [79]*.* We favor a model whereby interactions involve cross-linked PG peptides and the presence of an additional D-Ala results in steric hinderance, which impact the PG-p83/100 interaction(s). This scenario is somewhat reminiscent of Braun’s lipoprotein (Lpp), which is covalently attached to the alpha carboxylic acid through the L-center of mDAP [80,81]. The primary function of Lpp is to be a positional scaffold for the *E. coli* PG sacculus, relative to the outer membrane. The distance between the inner and outer membranes of *B. burgdorferi* is ~ 20 – 40 nm [13,37]. If the extended N-terminal alpha-helix in the predicted structure of p83/100 (~ 8 nm) along with a C-terminal membrane-associated amphipathic alpha-helix are correct, then one could envision it functioning as a tether between the sacculus and the outer membrane (S5A Fig). This could occur through protein-protein interactions in the outer membrane, or in the periplasm, but it is this unknown protein that is surface exposed and primarily responsible for mediating tissue residency in interarticular spaces and/or periarticular vascular beds (S5B Fig and see below).

Lyme disease-causing *Borreliae spp.* are known to exhibit tissue tropism, leading to diverse and distinct pathologies [5]. Initial studies of an isogenic mutant of *p83*/*100* indicated that ear and heart tissues were devoid of viable bacteria 3 weeks post-infection, as well as a marked reduction in bacterial load in infected ankle joints [59]. We found that the *dacA* mutant, which possesses less PG-bound p83/100 (Fig 5A–5C), had no apparent difference in ear or heart burden, but bacterial DNA in mouse ankles was significantly reduced (Fig 4). A simple explanation for this subtle discrepancy is the time point in which each were assessed (3 weeks versus 4 weeks post-infection, Fig 4). We also noticed differences in bacterial load 2 weeks post-infection, but none were significant (S2 Fig). Together, we speculate that the PG-p83/100 complex is important in stabilizing the cell envelope and that this association becomes critical in the viscous joint space environment. We also argue that p83/100 alone cannot mediate Lyme arthritis since joint inflammation is still attenuated when bacterial burden is similar (Figs 3, 4, and S4). Thus, we favor a model in which both PG-p83/100 interactions (along with unknown, surface-exposed protein(s) (i.e., tissue and disease tropism)) and PG architecture (inflammatory mediator of arthritis) are key players in the murine Lyme arthritis arena. In other words, p83/100 functions as a PAP that may indirectly impact the fitness of the spirochete in the joint environment by affecting the tissue-dependent context of outer surface protein expression while the chemical constituents and organization of the PG sacculus directly contribute to its inflammatory capacity [82,83]. Our attempts to uncouple these biologically linked processes (i.e., phenotypic change in PG chemistry, P83/100 interaction, and presence of DacA) by complementing the *dacA* mutant with either a wild-type or catalytically inactive allele have failed for unknown reasons, but these studies will be important to improve our understanding of this system and identify causal relationships. It will also be intriguing to determine if the synovial fluid of human Lyme arthritis patients, which contains PG and potentially other PAPs, has detectable levels of PG-p83/100 complexes as well [13–15,84].

The bespoke connection between the unusual *B. burgdorferi* PG, and the equally irregular Lyme spirochete cell envelope, continue to be elucidated. Conventional reasoning posited that *B. burgdorferi* does not produce PAPs. We now have three PAP candidates that are intertwined in the physiology, life cycle, and pathogenesis of *B. burgdorferi* (NapA, p83/100, and BpiP [85]). Further assessment of these proteins, their interactions, and the coordination thereof, will certainly further our understanding of Lyme disease and may provide new strategies to prevent or mitigate human illness.

## Materials and methods

### Ethics statement

All animal studies were approved by the Virginia Tech Institutional Animal Care and Use Committee (Protocol numbers 20-051 and 23-099).

### Bacterial strains and culture conditions

All strains used in this study were derived from the *B. burgdorferi* type strain B31 [10] The primary strain for all experiments was the infectious *B. burgdorferi* B31-5A3 [27], kindly provided by Dr. Jenifer Coburn (Medical College of Wisconsin), from which the B31-5A3/*dacA* strain was derived as described below. The *p83*/*100* (locus tag *bb0744*) mutant strain—*B. burgdorferi* B31-ML23/*bb0744*—was graciously provided by Dr. Jon Skare [59]. All strains used in this study were cultured in Barbour-Stoenner-Kelly II (BSK-II) medium, pH 7.6, supplemented with 6% heat inactivated rabbit serum (Pel-Freez Biologicals) at 34°C unless stated otherwise [86]. Organ outgrowth studies were conducted using the same media described above but supplemented with a final concentration of 2% of gelatin, and amphotericin (2.5 μg/mL), fosfomycin (20 μg/mL), and rifampicin (50 μg/mL). When necessary, the culture media was further supplemented with gentamicin (B31-5A3/*dacA*, 40 µg/mL) or streptomycin (B31-ML23/*bb0744,* 100 µg/mL).

To assess any potential growth defects, BSK-II (5 mL) was inoculated with 5 x 10^5^ cells and bacterial density was determined in triplicate every 24 hours using a 0.02mm Petroff-Hausser hemocytometer (Hausser Scientific) [87]. Data were fit to a log-linear model to determine the exponential growth phase and these values were used to calculate growth rate.

### Swarm plate assay

To assess whether the change in PG structural motifs has any impact on the motility of the B31-5A3/*dacA* strain, we prepared semi-solid medium plates as described previously with a final concentration of 0.5% (w:v) of low-melt agarose [20]. B31-5A3 and B31-5A3/*dacA* cultures (15 mL, 34°C) were harvested at mid-log and resuspended to a concentration of 10^9^ cells/mL. Three plates were inoculated on both sides with 7.5 µL of either suspension. After five days of incubation at 37 °C and 5% CO_2_, the radius of each colony was measured in five different directions and averaged.

### Genetic manipulation of *B. burgdorferi* and whole genome sequencing

Clone T08TC493 was previously described and contains a Himar*1* transposon insertion event 300 bp downstream of the predicted *bb0605* start site [24]. DNA primers were created (F: 5’-GGTCCAAAAAGAACATTTGAAACCG-3’; R: 5’-TCTTCTATGCTTTCAATGACCTGCT- 3’) to amplify Himar*1* and ~2kb of flanking DNA and the product was sub-cloned (TOPO TA pCR2.1). The resulting plasmid was purified, sequenced, then linearized and used to transform *B. burgdorferi* B31-5A3 by electroporation, and recombinant clones were selected as described (Samuels 1994). For whole genome sequencing, bacteria were cultured in 40 mL of BSK-II, harvested by centrifugation (4,000 x *g* for 10 mins) and washed with PBS before genomic DNA purification using Zymo Research Quick-DNA Miniprep kit following manufacturer’s recommended procedures. Whole genome sequencing was performed by the Microbial Sequencing and Analysis Center at Pittsburgh University (Pittsburg, PA), which resulted in > 410X coverage for each sample. Unicycler processed and assembled all sequence data, which was mined for polymorphisms and the Himar*1* integration site. Data were also mined to determine the plasmid profile of each strain, which was the same, and identical to previously published for our B31-5A3 clone [20]. One sequence confirmed clone was named *B. burgdorferi* B31-5A3/*bb0605* (or/*dacA)* and the focus of our studies.

### PG purification and analysis

0.25–0.5 L of mid-log cultures were subject to isolation procedures established previously [13,20,42]. Briefly, following mutanolysin-mediated peptidoglycan digestion, the isolated supernatant containing soluble muropeptide fragments was frozen and dried using a high vacuum line coupled with a liquid nitrogen solvent trap. The purified muropeptides were resuspended in 150 µL of saturated sodium borate buffer, pH 9.25. A 50 µL aliquot of 100 mg/mL NaBH_4_ was added dropwise and after 1 hr incubation the reaction was quenched with approximately 10 µL of formic acid (final pH 3). The samples were then snap frozen and dried once more with a high vacuum line. The dried muropeptides were resuspended in H_2_O:MeCN (200 µL, 9:1, v:v) containing 0.1% formic acid and sonicated in a water bath for 10 min followed by centrifugation at 13,000 x *g* at 4°C for 10 min. 180 µL of the supernatant was transferred to a LCMS vial for analysis.

### Microscopy

To determine whether zonal growth was impaired, both B31-5A3 parental, and B31-5A3/*dacA)* mutant strains were cultured to mid-log (10^7^ cells/mL) and incubated with 0.25 mM HADA for 4 hours, as previously described [42]. The cultures were then centrifuged at 3,500 x *g* for 15 minutes and washed three times with PBS. The final pellets were then resuspended in 100 µL of PBS and 5 µL of these suspensions were spotted on PBS agarose (2%) pads prior to imaging at RT with a Zeiss Axio Observer equipped with an oil-immersion phase-contrast objective Plan Apochromat 100 × /1.45 numerical aperture (Nikon) coupled to a Hamamatsu Orca-Flash 4.0 V3 Digital CMOS camera. Automated cell detection and cell-length measurements were facilitated by Oufti [88]. Demograph, signal intensity scatter plots, and population-level line scan analyses involved custom MatLab scripts, published elsewhere [15,42,43].

### Mouse studies

Three- to four-week-old C3H/HeJ mice were purchased from Jackson Laboratory. To manage the experimental procedures and data collection associated with the number of mice in this study, all data collected and shown are the results collated from two independent experiments. Both strains of *B. burgdorferi* were cultured to mid-log (5x10^7^ cells/mL) and resuspended to a concentration of 1x10^6^ cells/mL in PBS following centrifugation at 3,500 x *g* and 4 washes with PBS. Mice (*n* = 25 per strain of *B. burgdorferi*) were subcutaneously inoculated with either strain between the scapulae with 100 µL of this suspension; an inoculum of 1x10^5^ cells.

The mice were euthanized through CO_2_ asphyxiation followed by cervical dislocation and heart puncture at 2, 4, and 12 weeks post-inoculation. Tissues including the heart, ears, bladders, and tibiotarsal joint were harvested for outgrowth culture, qPCR quantification of bacterial load, and histopathology. Outgrowth cultures were monitored by dark-field microscopy for the presence of viable cells. Cultures were considered positive if motile spirochetes were present 4–6 weeks post inoculation.

### Arthritis scoring

All mice were examined on alternate days for signs of hindleg swelling and inflammation and assigned a score based on the severity of visible signs of arthritis using a scale and method previously described [47,48]. Briefly, no swelling of the carpal and tarsal joint or redness of the paw is a score of 0; some swelling accompanied with redness of the paw is a 1; moderate swelling, redness of the paw is a 2; severe swelling, redness of the paw, and occasional swelling of the interphalangeal joint is a 3.

### Histopathology and semiquantitative analysis

Rear limbs with the skin removed and previously immersion-fixed in 10% neutral buffered formalin were received blind to experimental manipulation by the Comparative Pathology Research Core (CPR Core), Department of Comparative Medicine, Yale University, School of Medicine, New Haven CT. The limbs were decalcified using Decal Solution (Richard Allen Scientific, Kalamazoo, MI) then trimmed and placed medial side down into tissue cassettes by routine methods. The tissue cassettes were submitted to the CPR Core Histology Laboratory for processing, embedding, sectioning and staining by hematoxylin and eosin (HE) and cover-slipped by routine methods.

The tissues were examined blind to experimental manipulation using the standard Barthold Lyme tenosynovitis (arthritis) semiquantitative scoring system [89]. Briefly, the knee and tibiotarsal joints were scored for arthritis severity on a scale of 0 (negative) to 3 (severe) in a blinded fashion as described previously by CJB [90–92]. The slides were examined using an Olympus BX53 light microscope, photographed using an Olympus DP 28 camera and Olympus cellSens Standard 4.2.1 software. The images were optimized and figures made using Adobe Photoshop 26.8.1.

### qPCR

DNA was isolated from murine tissue samples using the Quick-DNA Miniprep Plus Kit (Zymo Research). *B. burgdorferi recA* and mouse *nidogen* standard curves were generated using DNA isolated from B31-5A3 grown in culture and mouse tissue, respectively. These were quantified spectrophotometrically, from which the number of genome copies were determined [93].

qPCR was performed with Apex GREEN qPCR Master Mix (Genesee Scientific) on a CFX Opus 96 (BioRad) with the following parameters: 95°C for 15 minutes followed by 40 cycles (95°C for 15s, 59°C for 30s, 72°C for 30s). The primers used to amplify *B. burgdorferi*-specific *recA* were F: 5′-GTGGAT CTA TTG TAT TAG ATG AGG CTC TCG-3′ and R: 5′-GCC AAA GTT CTG CAA CAT TAA CAC CTA AAG-3′. The primers used to quantify mouse *nidogen* were F: 5′-CCA GCC ACA GAA TAC CAT CC-3′ and R: 5′-GGA CAT ACT CTG CTG CCA TC-3′. Data was analyzed through comparison to the standard curves and normalization to the reference gene [93,94].

### RT-PCR

Mid-log phase exponential, 40 mL cultures (10^7^ cells/mL) of *B. burgdorferi* B31-5A3 and B31-5A3/*dacA* were harvested at 3,250 x *g* at 4°C for 15 minutes and washed twice with PBS. RNA was purified and processed immediately using Zymo Quick-RNA Miniprep Plus Kit (Zymo Research) using the manufacturers recommended procedures for bacterial cells with one modification. After column purification, each RNA sample was treated with DNaseI for 20 minutes, re-purified, and then digested again with DNaseI (i.e., two 20-min digestions). After final purification, ‘qPCR’ was performed on each DNase-treated RNA sample to ensure preparations were free of DNA using *recA* primers listed above.

RT-PCR studies to assess expression of each target locus were performed on purified RNA from*B. burgdorferi* B31-5A3 and B31-5A3/*dacA* with the following primer pairs: *bb0605* (F: 5’- CTG GAT ATA GCA GCG AGA ATA AG-3’, R: 5’-GCA GTT CCT AAA TTT CTA CTC TTT GG-3’); *cheD* (F: 5’-CTT GGT TCT TGT GTT GCT GTT GTG C, R: 5’- CTT CCC CTT TGA TCA GGA GAT TAG TC-3’); *bb0607* (F: 5’-GCT TTA TGA AGA GAG GTT AAG GC, R: 5’-CTG ATC ATC ATC CCC AAC ACA ACA C-3’); *bb0608* (F: 5’- CCA ATT GAA ATT GTT GAA GAG GAT G-3’, R: 5’-CCC CAA GAT GAT AGC TAC TCC AAT C-3’). All reactions were performed on the same plate, using PCRBIO 1-Step Go RT-PCR Kit (PCR Biosystems) following the recommended procedures. Delta Cq values were acquired for each sample and target, then normalized by values attained by the constitutively expressed *flaB.* Melting curve analysis of each amplicon confirmed a single species was produced for each primer pair. The normalized, relative values attained were plotted from three technical replicates. We note *bb0608* was initially included into our analysis because we thought the locus was part of the same reading frame. After further examination, there appears to be sufficient space for an independent promoter, and our data suggest this promoter is not active in vitro (Fig 1B).

### Serology

B31-5A3 lysate was diluted to 2 µg/mL (Bradford assay) in carbonate coating buffer (90 mM NaHCO_3_ and 60 mM Na_2_CO_3_, pH 9.6), coated onto 96-well plates and incubated overnight at 4°C. After blocking with 100 µL SeaBlock (Thermo-Fisher) at 37°C for 2 hours, serially diluted mouse blood plasma samples (100 µL/well) were added to the wells and incubated at RT for 1 hour. Afterwards, HRP-conjugated Goat anti-Mouse IgG (1:25,000) was added to each well and incubated for an additional hour. Finally, 100 µL of 1-Step TMB ELISA Substrate Solution (Thermo Scientific) was added and incubated for 10 minutes. The reaction was stopped with 100 µL 1.5 N sulfuric acid and the end-point absorbance at 450 nm was measured for avidity determination.

### LCMS analysis

Analyses were performed as published previously [20] on a Shimadzu LCMS9030 QToF system coupled to a LC-40B X3 UPLC, a SIL-40C X3 autosampler (10°C), and a CTO-40C column oven (40°C). Borohydride-reduced mutanolysin-digested muropeptides were separated on a Waters BEH C18 column (2.1 x 50 mm, 1.7 µm particle size), at a flow rate of 0.4 mL/min. Solvent A was water and Solvent B was MeOH, with both solvents containing 0.1% formic acid (v/v). The initial solvent condition was 99:1 (A:B) for 3 min. This was followed by stepwise linear gradients first to 8% Solvent B (12 min), then to 20% Solvent B (24 min), and finally 95% Solvent B (25 min), followed by a hold at 5% B for an additional 5 mins (30 min total). The column was then re-equilibrated to 1% B (1 min of 1% B, followed by a 5 min re-equilibration time). The reduced muropeptides were subjected to electrospray ionization in positive ion mode using data dependent acquisition. Interface voltage was 4.0 kV at 300°C with a desolvation temperature of 526°C and a DL temperature of 250°C. Gas flows for nebulizing, heating, and drying gases were 2, 10, and 10 L/min, respectively. The *m/z* range for the precursor MS scan was 400–2000, with a fragmentation data range of 50–2000 and a collision energy of 35 ± 10 V.

### Western blots

Briefly, 50–100 mL of each strain was cultured to mid-log phase, pelleted by centrifugation at 3,500 x *g* for 15 minutes, and washed four times with PBS. Washed cell pellets were resuspended in 0.5 mL of PBS, sonicated, and the resulting crude lysate was stored at -80°C. Equal amounts of sonicated lysates, validated by a Bradford assay (Thermo Scientific), were separated by protein gel electrophoresis on SurePAGE Bis-Tris 4–20% precast gels (GenScript) and transferred onto PVDF membranes (Genesee Scientific). Immunoblots involved probing membranes with rabbit or rat antisera against FlaB (1:1,000), NapA (1:5,000), and p83/100 (1:5,000), which were kindly gifted by Melissa Caimano (UConn), Frank Gherardini (NIH), and Jon Skare (Texas A&M University), respectively. Western blots to detect OspA used antibodies (1:1,000) purchased from Rockland Inc. Rabbit IgG:HRP (Jackson Labs) was used to detect anti-NapA, and anti-OspA at dilutions of 1:12,000, and 1:16,000, respectively; rat IgG:HRP (Invitrogen) was used against anti-FlaB and anti-p83/100 at a dilution of 1:8,000. The membranes were incubated with SuperSignal West Pico PLUS chemiluminescent substrate (Thermo Scientific) and imaged with an Odyssey M (LI-COR).

### PAP studies

PG was purified from 250 mL bacterial cultures, as described above, but stopped prior to chymotrypsin step. Prior to protease digestion, sacculi were resuspended in 500 µL of PBS and split in half. One 250 µL sample (-PK) was stored at 4°C while the other 250 µL sample was first treated with alpha chymotrypsin, as described previously [15,20], then 10 µg/mL of proteinase K overnight. Proteinases were inactivated and PG purified afterwards, as described above.

Proteinase treated and untreated sacculi, from each strain, were assessed using ELISA and Immunofluorescence. ELISA studies involved two separate procedures: PG quantification and p83/100 quantification. The former was performed as reported elsewhere [14]. p83/100 ELISA involved coating microtiter plates with 1 µg/mL of r-mAb2G10 to immobilize each PG preparation. Unbound material was removed with three washes of PBS-T (PBS + 0.05% Tween-20), and sacculi were blocked for 2 hours at 37°C using SeaBlock (Thermo Fisher). Excess blocking agent was removed with 2 washes of PBS, and sacculi were probed for p83/100 using rat anti-p83/100 serum (1:25,000). Non-specifically bound antibody was removed with 4 washes of PBS-T, anti-rat IgG:HRP (Jackson labs, 1:30,000), was used to detect the p83/100 polyclonal antibody, which was followed by 4 additional PBS-T washes and TMB one-step ultra (Thermo Fisher) substrate for colorimetric enzyme detection. Immunofluorescent studies were performed exactly as described previously [15], but rat anti-p83/100 (1:1,000) and rat anti-IgG:Alexa647 (1:400) antibody pairs were used.

### Statistical analysis and modeling

All statistical analyses are described throughout the text and figure legend, which were performed using GraphPad Prism 10. AlphaFold 3.0 was used for all protein structural modeling [95].

## Supporting information

S1 Table Summary of whole genome sequence data of *dacA* mutant strain. (PDF)

S2 Table A. Peptidoglycan cross-linking analysis; B. Analysis of peptidoglycan containing di-D-Alanine cross-links.
(PDF)

S1 Fig Predictive structure and growth kinetics of *dacA* mutant strain. (TIFF)

S2 Fig Bacterial load and serology of animals two-weeks post infection. (TIFF)

S3 Fig Single example of murine Lyme arthritis caused by *dacA* mutant strain. (TIFF)

S4 Fig Bacterial load in ankles 12 weeks post-infection. (TIFF)

S5 Fig Predicted 3-dimensional structure of p83/100 and model of cell envelope. (TIFF)
