# Supplementary material for: Peptidoglycan architecture dictates protein interactions, tissue tropism, and arthritis in the Lyme disease spirochete Borrelia burgdorferi
Source: PLoS Pathog. 2026 Jan 20;22(1):e1013849. doi: 10.1371/journal.ppat.1013849 (PMC12818604; doi:10.1371/journal.ppat.1013849)
Supplement: S1 Table — (PDF) [file ppat.1013849.s001.pdf]

Table S1. Summary of whole genome sequence polymorphisms present in *B. burgdorferi* B31-5A3/*bb0605* mutant, relative to *B. burgdorferi* B315A3 parental control strain*. Note that Tn insertion site (+300, relative to start site) of the *bb0605* mutant strain is not shown for simplicity.

Mutation	Event	Coordinate	Location	Result
I	+C	3,140	BB003 (pseudo)	—
S	T→C	18,469	BB0020	V→A
S	T→A	56,155	BB0059	Silent
S	T→A	63,111	Intergenic	—
D	-A	75,017	BB0078 (pseudo)	—
I	+A	138,870	BB0140 (pseudo)	—
I	+A	138,881	BB0140 (pseudo)	—
S	T→C	203,019	BB0202	Silent
S	T→A	210,286	Intergenic	—
I	+A	311,632	Intergenic	—
D	- G	366,107	BB0357 (pseudo)	—
I	+T	366,152	BB0357 (pseudo)	—
S	T→C	390,075	BB0380	Silent
I	+AT	438,324	Intergenic	—
I	+T	747,897	BB0710 (pseudo)	—
I	+A	757,751	Intergenic	—
I	+A	862,670	BB0815 (pseudo)	—

I: Insertion; D: Deletion; S: Substitution

Pseudo: pseudogene

*Parental strain reference sequenced in DeHart et al., 2021 [20]
