# Supplementary material for: Peptidoglycan architecture dictates protein interactions, tissue tropism, and arthritis in the Lyme disease spirochete Borrelia burgdorferi
Source: PLoS Pathog. 2026 Jan 20;22(1):e1013849. doi: 10.1371/journal.ppat.1013849 (PMC12818604; doi:10.1371/journal.ppat.1013849)
Supplement: S2 Table — Peptidoglycan cross-linking analysis; B. Analysis of peptidoglycan containing di-D-Alanine cross-links. (PDF) [file ppat.1013849.s002.pdf]

Table S2A. Muropeptides participating in cross-linking.

Strain	∑Crosslinked PG peak area	∑PG peak area	% crosslinked
B31-5A3	6407946	19544760	32.79
B31-5A3/*bb0605*	5962560	17706889	33.67

Table S2B. Muropeptides participating in cross-linking containing di-D-Ala.

Strain	∑Crosslinked di-D-Ala peak area	∑Crosslinked PG peak area	% di-D-Ala crosslinked
B31-5A3	n.d.	6407946	n.d
B31-5A3/*bb0605*	3312890	5962560	55.56

n.d. none detected
