# Supplementary figures and images for: Peptidoglycan architecture dictates protein interactions, tissue tropism, and arthritis in the Lyme disease spirochete Borrelia burgdorferi
Source: PLoS Pathog. 2026 Jan 20;22(1):e1013849. doi: 10.1371/journal.ppat.1013849 (PMC12818604; doi:10.1371/journal.ppat.1013849)

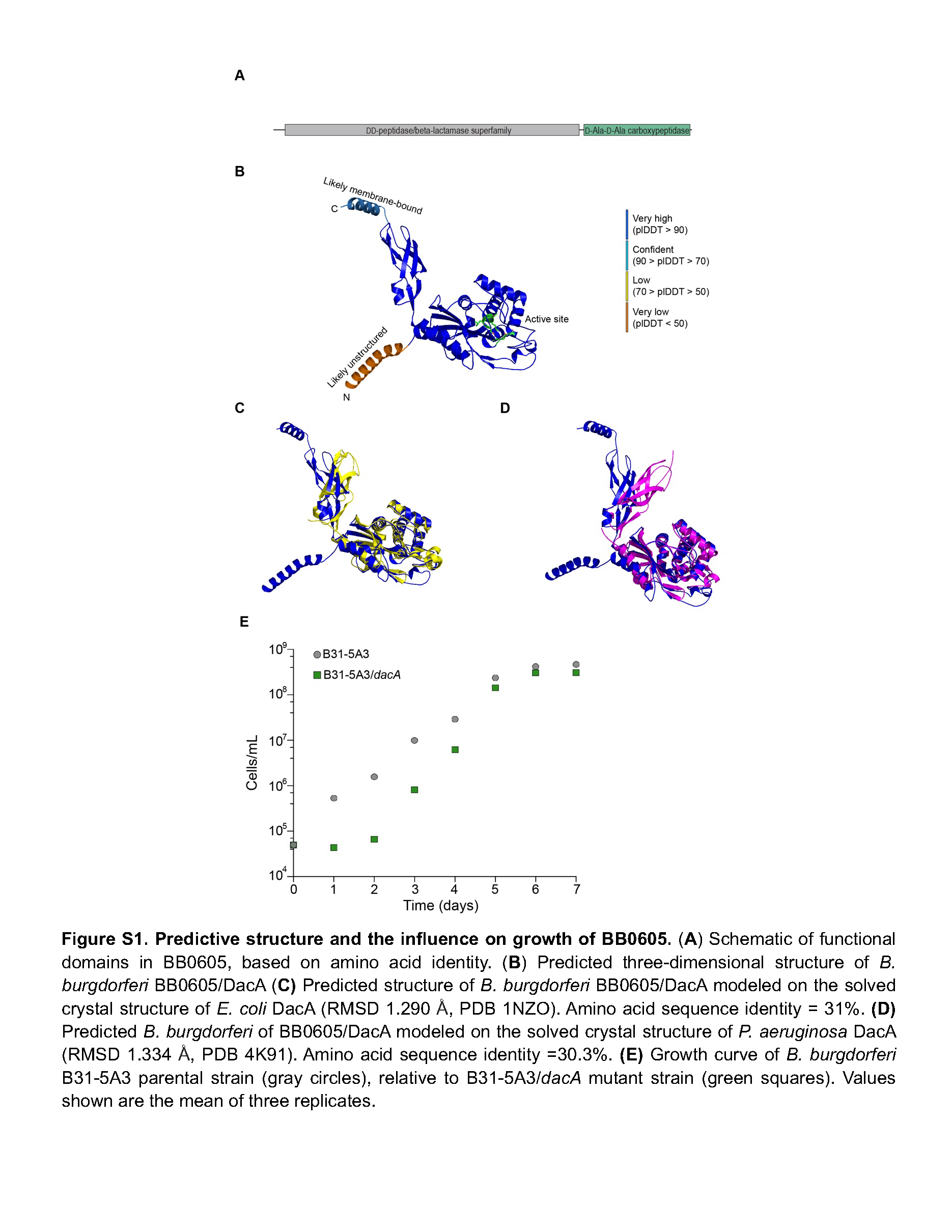

Supplement: S1 Fig — (TIFF) [file ppat.1013849.s003.tiff]

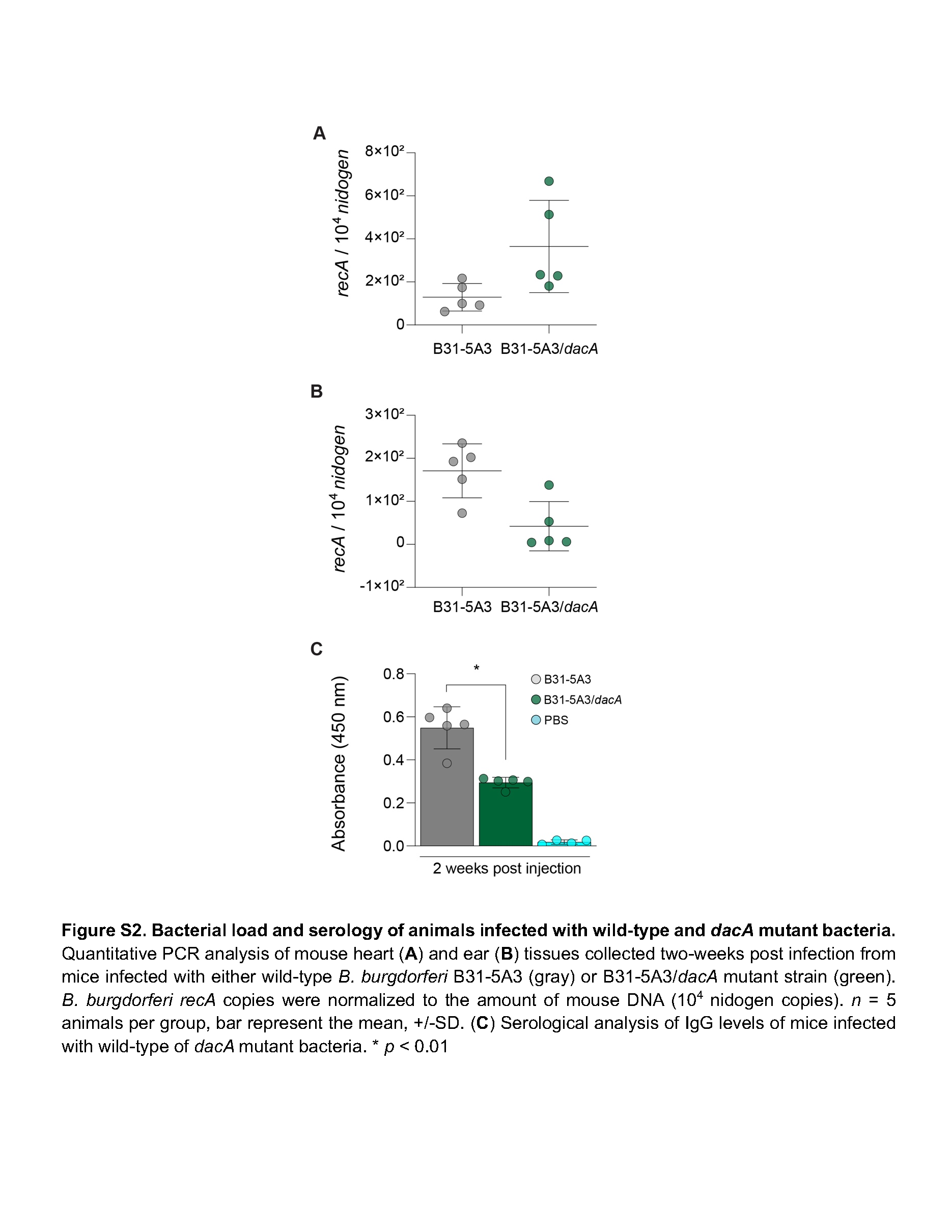

Supplement: S2 Fig — (TIFF) [file ppat.1013849.s004.tiff]

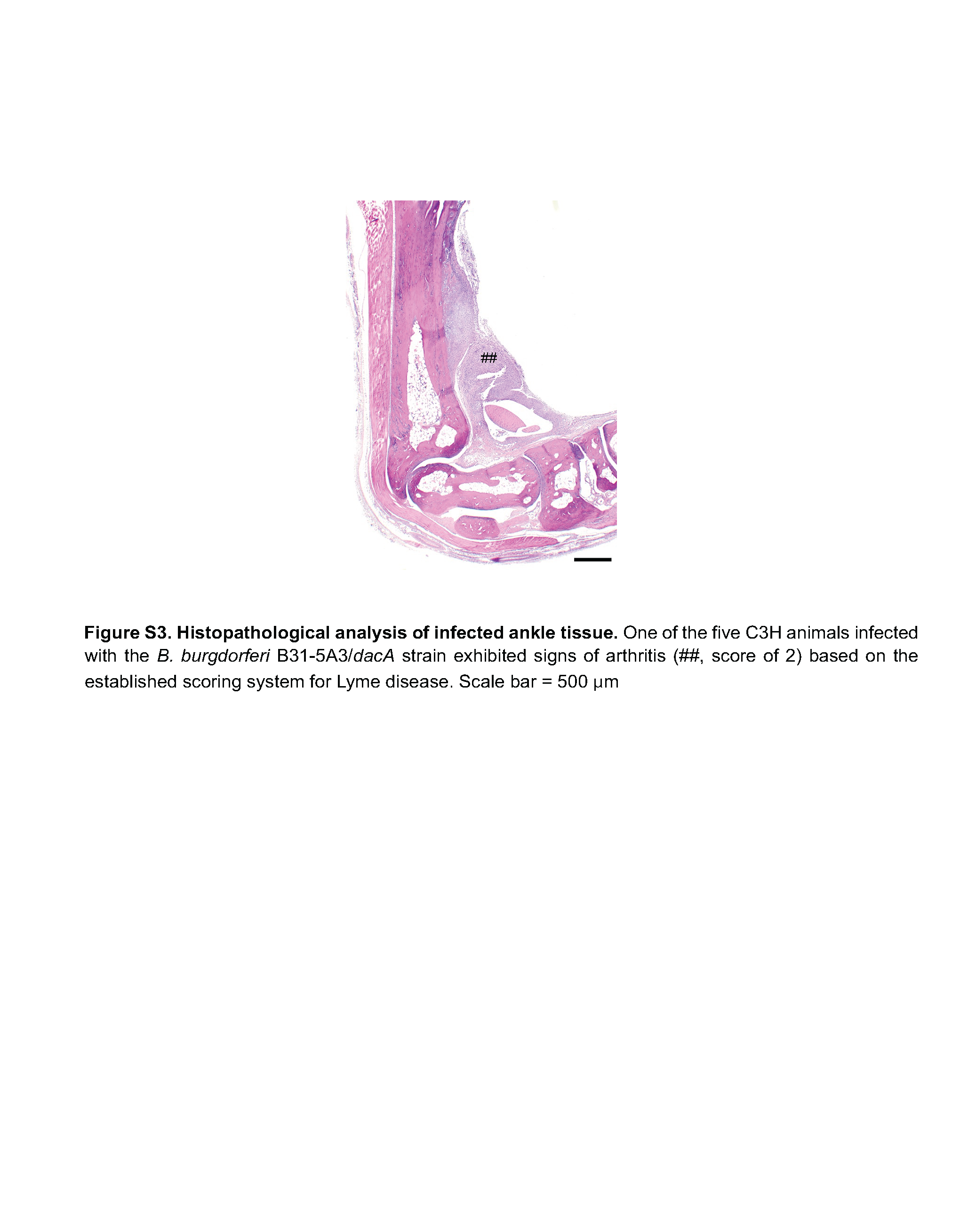

Supplement: S3 Fig — (TIFF) [file ppat.1013849.s005.tiff]

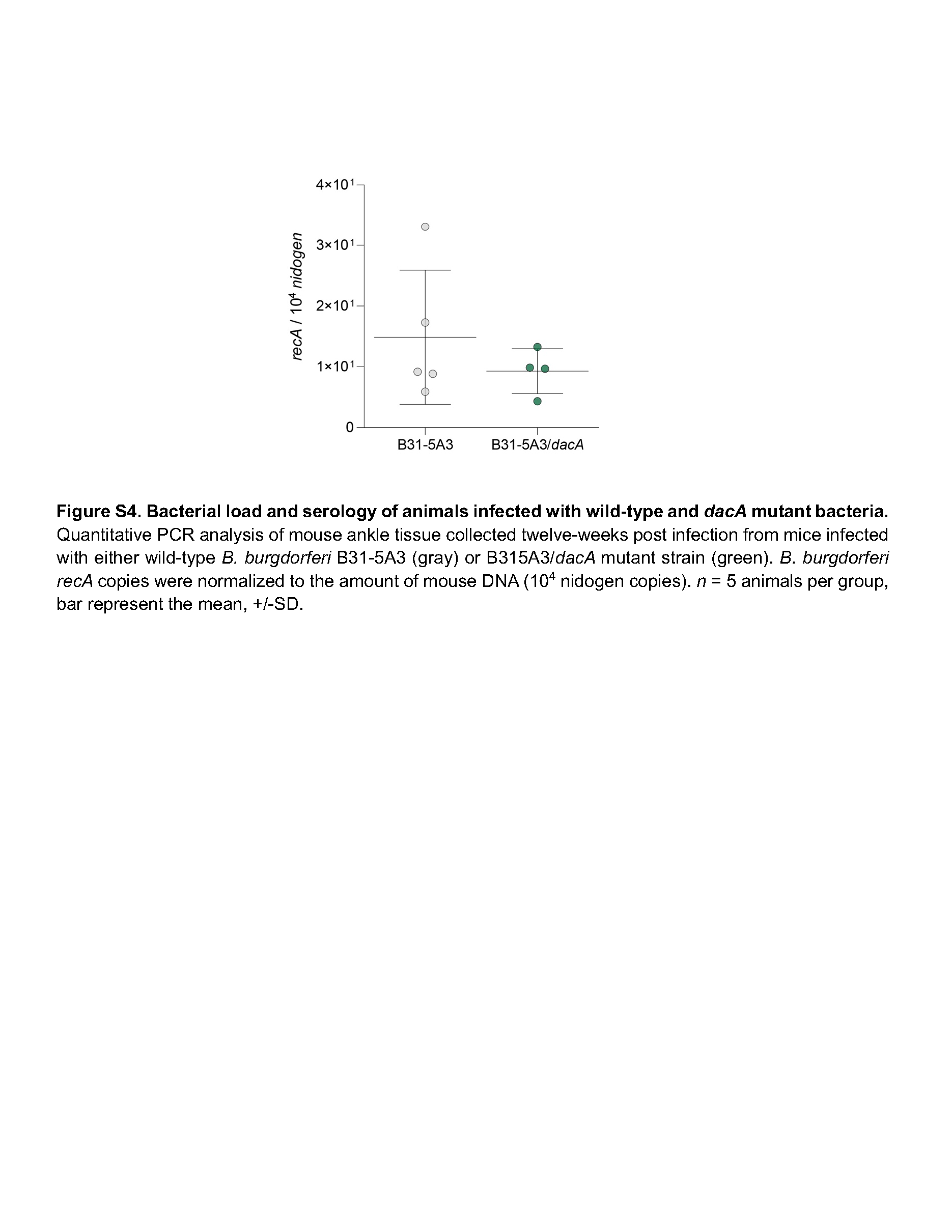

Supplement: S4 Fig — (TIFF) [file ppat.1013849.s006.tiff]

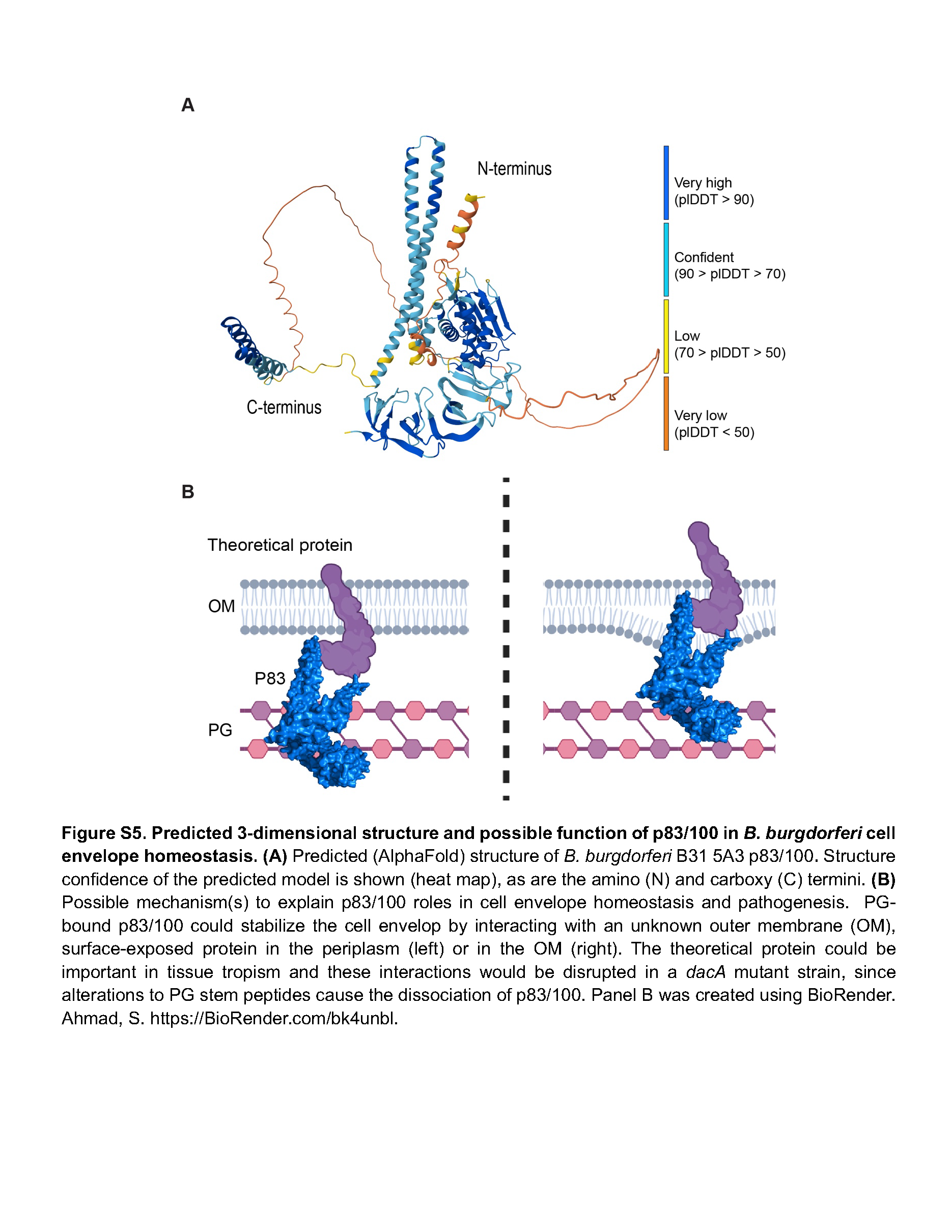

Supplement: S5 Fig — (TIFF) [file ppat.1013849.s007.tiff]
